# The psychosomatic impact of Yoga in medical education: a systematic review and meta-analysis

**DOI:** 10.1080/10872981.2024.2364486

**Published:** 2024-06-11

**Authors:** Sabyasachi Maity, Raman Abbaspour, Stephan Bandelow, Sehaj Pahwa, Taraneh Alahdadi, Sharan Shah, Praghosh Chhetri, Ameet Kumar Jha, Shreya Nauhria, Reetuparna Nath, Narendra Nayak, Samal Nauhria

**Affiliations:** a Department of Physiology, Neuroscience, and Behavioral Sciences, St. George’s University School of Medicine, True Blue, Grenada; b Department of Electrical Engineering and Computer Science, York University, Toronto, Canada; c Medical Student Research Institute, St. George’s University School of Medicine, True Blue, Grenada; d Department of Physiology, St. Matthew’s University School of Medicine, Georgetown, Cayman Islands; e Department of Anatomical Sciences, St. Matthew’s University School of Medicine, Georgetown, Cayman Islands; f Department of Child Protection, Cayman Islands Red Cross, Georgetown, Cayman Islands; g Department of Education Service, St. George’s University School of Medicine, True Blue, Grenada; h Department of Microbiology, St. Matthew’s University School of Medicine, Georgetown, Cayman Islands; i Department of Pathology, St. Matthew’s University School of Medicine, Georgetown, Cayman Islands

**Keywords:** Yoga, medical student, systematic review, medical education, meta-analysis, physiology, Anxiety, stress, academic performance

## Abstract

Non-clinical approaches such as meditation, yoga, and mindfulness are popular traditional therapeutical interventions adopted by many educational institutions to improve the physical and mental well-being of learners. This study aimed to evaluate the effectiveness of yoga intervention in improving cardiopulmonary parameters such as blood pressure, heart rate, pulmonary function tests and psychosomatic symptoms such as depression, anxiety and stress in medical and dental students. Using the PRISMA protocol, a search from databases such as PubMed, Scopus, and Embase resulted in 304 relevant articles. After screening the title and abstracts, 47 papers were analyzed thoroughly and included in the qualitative analysis. 18 articles with homogenous statistical data on physiology and psychological parameters were included for meta-analysis. In comparison to the control group, the study showed a significant reduction of systolic blood pressure (SBP: 6.82 mmHg, z = -3.06, *p* = 0.002), diastolic blood pressure (DBP: 2.92 mmHg, z = -2.22, *p* = 0.03), and heart rate (HR: 2.55 beats/min, z = -2.77, *p* = 0.006). Additionally, data from 4 studies yielded a significant overall effect of a stress reduction of 0.77 on standardized assessments due to the yoga intervention (z = 5.29, *p* < 0.0001). Lastly, the results also showed a significant (z = -2.52, *p* = 0.01) reduction of 1.2 in standardized anxiety tests in intervention group compared to the control. The findings offer promising prospects for medical educators globally, encouraging them to consider reformation and policymaking in medical curricula to enhance academic success and improve the overall quality of life for medical students worldwide.

## Introduction

Yoga, an ancient practice promoting holistic well-being, has gained global popularity for its integration of body, mind, and breath [1]. It offers health benefits, stress reduction, and improved quality of life, aligning tradition with modern needs [2]. Yoga’s significance in modern times stems from its comprehensive approach to health and wellness, positively impacting physical fitness, mental clarity, stress reduction, emotional regulation, self-awareness, and overall well-being [2,3]. Activity- and occupation-based interventions like sports and yoga effectively address mental health, behavior, and social participation concerns in children and youth [4]. Mind-body exercises like yoga can be effective alternatives for stress reduction, as they modulate sympathetic-vagal balance [5]. Implementing mind-body physical activity, including yoga, in educational settings significantly improves stress-related physiological health markers in students [6]. Self-care interventions, such as stress management courses and mind-body techniques like yoga and meditation, effectively reduce perceived stress in graduate students, empowering them to manage stress in their future healthcare roles [7]. Incorporating appropriate aerobic exercise, including yoga, alleviates anxiety and enhances overall physical and mental health [8].

Medical students face significant challenges and pressures in today’s demanding world, resulting in high stress levels, burnout, and reduced well-being [9,10]. Recognizing the importance of addressing these issues, integrating yoga practices into medical education has emerged as a potential solution to promote students’ health and well-being [11]. Horiuchi et al. found that yoga benefits medical students and recommend its integration into academic health centers, fostering student wellness and empowering future physicians [11]. Yoga offers various benefits to medical students, improving physical well-being, preventing musculoskeletal issues, reducing stress, and enhancing focus and emotional resilience [12]. Yoga interventions present cost-effective options for stress reduction and musculoskeletal pain relief [13]. Yoga techniques and mind-body medicine consistently reduce stress and burnout in healthcare workers exposed to heavy workloads and high stress levels [14].

While supported by various studies and anecdotal evidence, conducting a comprehensive meta-analysis will provide a rigorous evaluation of the impact of yoga on medical students’ well-being and academic performance. This meta-analysis aims to systematically review the literature on the impact of yoga practices on medical students’ physical and psychological well-being. This paper reviews the evidence that the yoga leads to improvement of blood pressure (BP), heart rate (HR), stress and anxiety levels, academic performance, and overall quality of life. Thus, contributing to a comprehensive understanding of the potential benefits and implications of integrating yoga into medical education [11]. Ultimately, this study aims to guide educators, administrators, and policymakers in implementing effective interventions for the well-being of medical students in the contemporary educational landscape.

## Methods

### Search strategy

Preferred Reporting Items for Systematic Reviews and Meta-Analyses (PRISMA) and Meta-Analysis of Observational Studies in Epidemiology (MOOSE) protocols were followed by the researchers [15,16]. The protocol used in this study was registered in International prospective register of systematic reviews (PROSPERO), the Center for Reviews and Dissemination, University of York (CRD42023429107) prior to the commencement of the project.

Published studies were searched in electronic databases namely, PubMed (US National Library of Medicine, National Institutes of Health), Scopus, Embase, and google scholar for potentially relevant studies from inception up to December 2022. Articles published in English from selected databases were included only. The authors agreed among themselves on the final search strategy. The medical subject headings (MeSH) search terms included ‘yoga,’ ‘medical students’ and ‘dental student’ including all subheadings.

### Selection of studies

Three reviewers (SS, SP and TA) independently evaluated the papers retrieved based on their titles and abstracts. To be included for a detailed examination, the relevant papers had to meet the following criteria. They reported data on the prevalence, impact of yoga intervention or practice in medical or dental students at the undergraduate level and conducted in any geographic location. All types of studies, both qualitative and quantitative study designs, including cross-sectional and cohort-based designs, were considered. Non-peer-reviewed materials such as editorials, letters, commentaries, incomplete data, reviews, conference posters, preprints, and dissertations were excluded from the analysis. Any uncertainties or disagreements regarding study selection were resolved through consensus among the reviewers.

### Data extraction

The data extraction process involved reviewer (RA, SM) gathering relevant information, which was then crosschecked by other researchers (AJ, SB). An Excel sheet was used to record details such as author names, publication year, geographical location, study duration, age range of participants, sample size, Yoga intervention type, and reported benefits of yoga for each eligible study. In case of incomplete data or unclear aspects, attempts were made to contact the original study authors for clarification. Any discrepancies or disagreements among the reviewers was resolved through consensus and discussion with another reviewer (SN).

### Within-group mean difference variance estimates

The effect estimates for all outcomes are based on the difference between change over time in the intervention group compared to the same change in the control group. Accordingly, only studies that include control groups are included in the quantitative meta-analysis. We also list intervention effects found in studies that did not include control groups in the forest plots, since the consistency and direction of intervention effects is relevant to the conclusions, but these studies are not included in the overall effect estimates of the meta-analysis.

Change over time within each treatment group was presented in virtually all included studies as the outcome measure means and standard deviations in each group, before and after intervention. However, the meta-analysis requires the mean difference per group over time, and its standard deviation between the two time points. The mean difference is easily calculated by subtracting the group means at time 1 from time 2, but the standard deviation (SD) of this difference score is more difficult to estimate. In the case of unrelated samples, the SDs could simply be pooled via one of the standard pooling methods. However, repeated scores from the same group are related samples, where one would expect individual scores to correlate over time, reducing the expected variance compared to unrelated samples. This within-subject correlation is equivalent to the test-retest reliability of each outcome measure, which we used to estimate the within-group variances of the mean difference scores according to the well-known formula for the variance of the difference between related samples:

$$\mathrm{VAR}\left(X-Y\right)=\mathrm{VAR}\left(X\right)+\mathrm{VAR}\left(Y\right)-2*\mathrm{SD}\left(X\right)*\mathrm{SD}\left(Y\right)*\mathrm{Corr}\left(X,Y\right)$$

Within-subject test-retest correlations for cardio-vascular function parameters were assumed to be 0.95 for heart rate, 0.86 for systolic blood pressure (SBP), and 0.85 for diastolic blood pressure (DBP), as reported by [17]. Test-retest correlations for lung function parameters were taken from [18], averaged across the pediatric and adult cohorts. A correlation of 0.97 was assumed for all lung function outcomes, except functional vital capacity (FVC), for which 0.94 was reported. Test-retest correlations for the 10 item perceived stress scale (PSS) were averaged from all available studies with 1–4 week testing intervals listed in [19], arriving at an estimate of 0.77. The stress subscale of the 42-items DASS was used to assess perceived stress in one of the included studies, for which [20] report a test-retest reliability of 0.82. Test-retest correlations for the STAI state anxiety measure [21] range more widely across various populations and re-test intervals. We averaged estimates from studies with retest intervals close to 2 weeks, arriving at an approximate and conservatively estimated test-retest correlation of 0.75 [22]. One study assessed anxiety via the DASS anxiety subscale, for which we assumed a retest correlation of 0.86 according to [20].

To determine the sensitivity of the reported results to the test-retest correlation estimates described above, we repeated all analyses with lower assumed test-retest correlations, by subtracting 0.2 from the *r* values listed above (i.e., reduction by an absolute 20%). The resulting *r* values ranged from 0.55–0.77, at the low end of what would be considered a valid and reliable test. There was no change in the pattern of results, all effects that were previously significant remained so. Accordingly, the reported results appear robust even if test-retest correlations were significantly weaker than those reported in the literature.

## Statistical analyses

All analyses were carried out in R version 4.3.0, using package ‘meta’ for the meta-analyses, forest and funnel plots. For the physiological outcomes (cardio-vascular and lung function) we report the raw mean differences, because they are inherently meaningful and easier to interpret than a standardized mean difference. All included studies also used the same outcome scale for the physiological parameters, e.g., mm Hg for blood pressure or litres for forced expiratory volume. However, the psychological outcome scores rely mostly on questionnaires, which can have different scales according to the number of included items per domain, and different ranges of the Likert scale for each item. The varying scales make a 1-point change difficult to interpret, and impossible to combine without standardization. Accordingly, effects for the psychological outcomes are reported as standardized mean differences (SMD), essentially dividing the raw effect size by the standard deviation, akin to Cohen’s d effect sizes. We used Hedges’ g method for SMD estimates throughout, which includes an additional correction for small sample sizes compared to Cohen’s d.

## Results

### Search results and study characteristics

**Figure 1.**
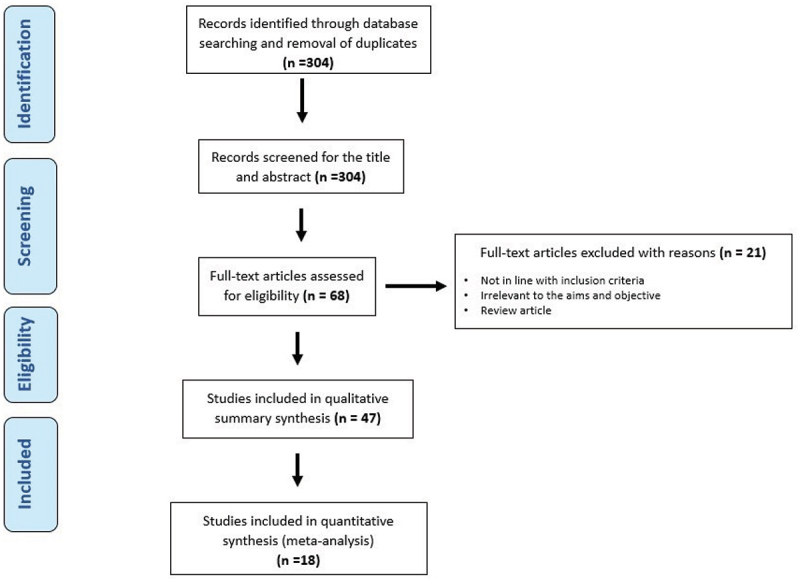
PRISMA protocol of literature search process.

In total, our initial search yielded 304 results. This includes both database searching and hand search. After reading the abstracts, 236 were excluded from this list. A further 21 were excluded once the entire article was thoroughly read. Overall, 47 articles were used for systematic review, a further 18 were included in the meta-analysis. One article with duplicate data was removed (Solanki, 2020). Figure 1 shows the PRISMA chart.

The exclusion criteria included articles not relevant to the objectives of this study, not conducted on medical students, full text not available even after contacting the authors, study design not an original research and articles where appropriate information and statistical data was not available. A detailed synthesis of the studies included is provided in Table 1.

**Table 1.** Summary of the 47 articles included in this study.

Author	Location	Population	Period of intervention	Conclusion
Jahan et al., 2020a [23]	Bangladesh	100 medical students (18–20 years)	10 min/day, 4 weeks	Yoga significantly improved respiratory parameters
Jahan et al., 2020b [24]	Bangladesh	100 medical students (18–20 years)	10 min/day, 4 weeks	Yoga led to a decrease in SBP, DBP and HR
Akhani et al., 2019 [25]	India	300 medical students > 18 years	6 days/weekly, 4 weeks	Yoga improves cardiorespiratory efficiency
Karthik et al., 2014 [26]	India	50 medical students (17–19 years)	30 min/day, 2 months	Yoga improves pulmonary functions
Pal et al., 2014 [27]	India	85 med students	1 h/day, 6 weeks	Right nostril breathing increases sympathetic activity. Left nostril breathing increases parasympathetic activity and promotes cardiovascular health.
Hm et al., 2012 [28]	India	60 med students (average 17.2 years)	N/A	Better sleep in Yoga groups.
Parshad et al., 2011 [29]	N/A	64 med student (21.3 ± 2.6 years)	6 weeks	Improves cardiovascular function. Proposes improvement in stress management (not supported by results)
Gopal et al., 2011 [30]	India	60 female medical students (17–20 years)	35 mins/day, 12 weeks	Yoga decreases physiological, autonomic, endocrine and psychological effects of examination stress.
K et al., 2017 [31]	India	100 med students (18.16 ± 0.38 years)	90 Days	Yoga reduces HR, SBP and DBP
Bharati, 2016 [32]	India	58 female med student	1 h/day, 5 day/week, 3 months	Yoga reduces PMS symptoms better compared to calcium carbonate
Lakshmi and Vaithianathan, 2022 [33]	India	30 (18–25 years)	12 weeks	Yoga significantly decreases HR and improved aggression
Chhetri et al., 2020 [34]	India	52 male med student (20 Yoga; 20 meditation)	20 mins	No significant change in cardiovascular parameters between Yoga and Meditation groups.
Thirupathi and K, 2016 [35]	India	60 male med students	45 min daily for 10 weeks	Yoga improves lung function
Mehta et al., 2018 [36]	India	36 med students	1h daily for 4 weeks	Yoga significantly lowers SBP and DBP
Monson et al., 2017 [37]	USA	77 Dental students	60 min biweekly for 13 weeks	Yoga reduces pain
Hc et al., 2015 [38]	India	60 med students	Yoga group has been practicing yoga for 1 year	Yoga for 1 year could reduce physiological arousal
Pandey et al., 2016 [39]	India	40 med students	1h daily, 3 months	Yoga decreases salivary cortisol, which could be used to manage stress
Lee et al., 2022 [40]	USA	64 med students	8 yoga sessions	Yoga reduced stress while not impacting exam score
Ahlers et al., 2021 [41]	USA	53 med students	20 hours of yoga in total	Virtual Wellness and Learning Communities program promotes medical student wellness
Nakashima et al., 2020 [42]	USA	108 med students	2, 30 min sessions	Yoga improves wellbeing, reduces burnout
Waechter et al., 2021 [43]	Grenada/USA	44 med students	12 weeks	No significant difference between Yoga or control group on stress, anxiety, psychological distress or academic performance
Nithiya and Palve, 2018 [44]	India	250 med students	1 month	significant decrease in anxiety, but not stress following yoga
Bansal et al., 2013 [45]	India	90 med students	45 min/day, 1 month	significant improvement in somatic symptoms, anxiety, social dysfunction, and depression following yoga.
Prasad et al., 2016 [46]	USA	27 med students	6 weeks	Yoga reduces stress and increases feelings of peace, focus and endurance
Simard and Henry, 2009 [47]	Canada	16 med students	16 weeks	Yoga decreases stress and improves general well-being
Shankarapillai et al., 2012 [48]	India	100 dental students	1 week	Yoga reduces stress
Sunita et al., 2022 [49]	India	105 female med students	40 min, 12 weeks	Yoga decreases anxiety, depression, and anger while increasing sense of wellbeing
Bond et al., 2013 [50]	USA	27 med students	Once/week, 11 weeks	Yoga increasing self-regulation and self-compassion. While not significant, it decreases perceived stress while increasing empathy
Erogul et al., 2014 [51]	USA	57 med students	20 min/day, 8 week	Yoga decreased perceived stress and increased self-compassion
Mehta and Taneja, 2013 [52]	India	36 med students	1h/day, 4 weeks	Yoga increases general well-being
Kukade and K, 2022 [53]	India	64 med students	1 h/day, 3 months	Yoga significantly improves happiness, psychological wellbeing, mindfulness, spiritual well-being, and sleep.
Alire et al., 2020 [54]	USA	77 dental students	90 min, for 10 weeks	Yoga significantly decreased stress-related symptoms
Singh et al., 2020 [55]	N/A	120 female dental students	40 min/day for 4 weeks	Yoga reduces stress. But yoga combined with motivational videos has a more substantial effect
Shapiro et al., 1998 [56]	USA	73 med students	2.5 h/week, 7 weeks	yoga group reported less depression, state anxiety, trait anxiety, GSI, and increases in empathy as well as spirituality
Danilewitz et al., 2016 [57]	Canada	30 med students	8, 1–1.5 hour weekly sessions	No significant result was found. However, it was suggested that peer-led yoga could improve wellbeing
Sreenivas et al., 2022 [58]	India	60 med students	30h of practice	Yoga improved attention and mental focus. It also could enhanced academic performance
Santhanam Kumar et al., 2020 [59]	India	20 med students	15 min	Yoga improved memory but had no significant effect on attention, processing speed, executive function or cognitive function.
Thomas and D, 2017 [60]	India	40 male med students	1 month	Yoga improves attentional control and working memory.
Kondam et al., 2017 [61]	India	80 med students	6 months	Yoga significantly improves memory, language fluency and visuospatial skills
Braun et al., 2019 [62]	USA	132 dental and dental hygiene students	1h	Yoga increases state mindfulness for students with high level of stress
P et al., 2018 [63]	India	100 med students	30 min, 5 days/week, 12 weeks	Yoga improves attention, concentration, and memory
Premalatha et al., 2021 [64]	India	73 dental students	6 days/week, 3 months	Yoga promotes academic performance
Shrestha et al., 2022 [65]	Nepal	176 med students	N/A (Questionnaire only)	Practicing yoga could help students to management their stress, increase their attention, and maintain physical and mental health.
Ankamreddy et al., 2019 [66]	India	150 med students	N/A (Questionnaire only)	Students have a positive outlook toward yoga but lack motivation.
Deshpande and Chari, 2016 [67]	India	116 med, 42 dental, 21 physiotherapy and 89 nursing students	2h/day, 5 days	Short term yoga practice did not significantly affect the perception of students toward the benefits of this activity
Nadig and Shah, 2020 [68]	India	206 dental students	N/A (Questionnaire only)	Wile 92% of students were aware of the benefits of yoga, only 35% practiced it regularly

**Figure 2.**
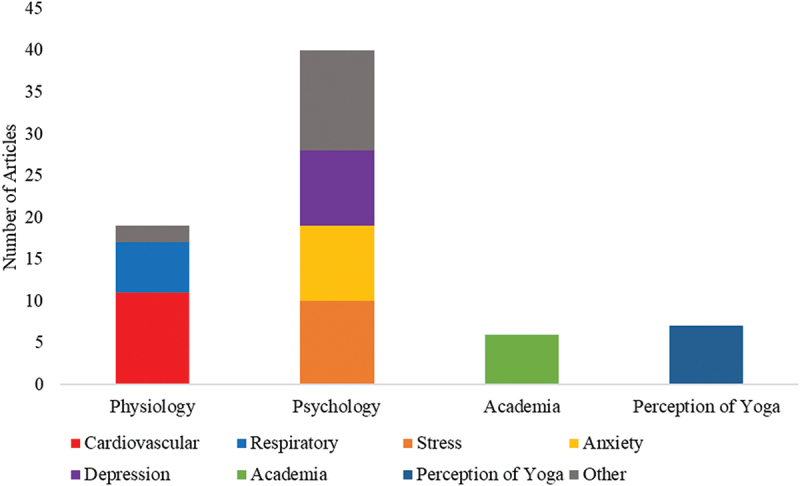
Distribution of Articles Across Investigation Categories. A total of 47 articles were included in our analysis.

The results of this study were categorized into four themes: Knowledge and awareness of yoga, effect of yoga on physiology, psychology and academia. Figure 2 represents the number of articles found in each of these four categories and their subcategory.

### Knowledge and perception of yoga

How students perceive the effectiveness of yoga could affect their commitment to this activity. Seven articles focused on the perception, knowledge, and attitude of students toward yoga. It was determined that more than 90% of students are aware of the benefits of yoga [65,65,67,68]. Despite this, on average, less than 20% of students practice yoga regularly [46,47,65,66,68]. The main barriers in practicing yoga were time management, lack of motivation, and laziness [66,67]. Overall, one study found that 95% of participants wish to practice yoga following the study [43]. Another study found that more than 90% of students believe yoga should be part of medical school’s curriculum to help them maintain physical and mental health [65].

### Physiological impact of yoga

#### Cardiopulmonary Improvement

**Figure 3.**
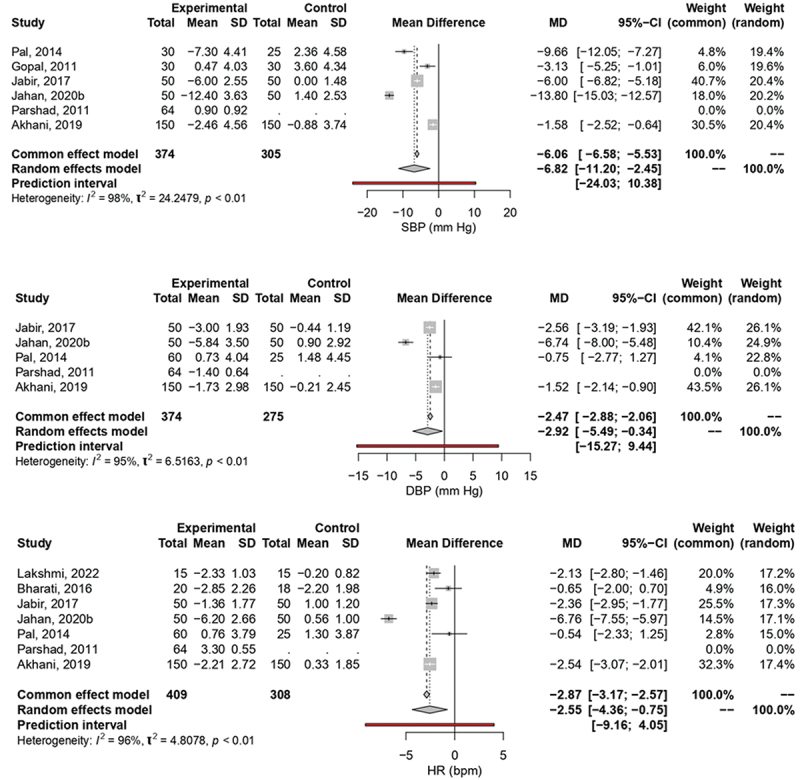
The Meta-analysis on the Effect of Yoga on systolic SBP (A), DBP (B), and HR (C) Among Medical and Dental Students.

Figure 3A summarizes the effects of yoga intervention on SBP. Data from five studies with more than 300 participants in treatment and control groups was included, yielding a significant overall effect of a SBP reduction of 6.82 mmHg due to the yoga intervention (z = −3.06, *p* = 0.002). The random effects model was chosen for the overall effect analysis due to the presence of significant variance between the included studies (I^2^ = 98.4%, *p* < 0.0001). Similarly, Figure 3B represents the effect of Yoga on DBP. Four studies with 310 and 275 participants in the intervention and control groups were included. Due to high variance (I^2^ = 95%, *p* < 0.0001), the random effects model was chosen for the overall effect analysis. The results showed significant (z = −2.22, *p* = 0.03) reduction of −2.92 mmHg in DBP following in intervention group compared to control. Lastly Figure 3C illustrates the effect of Yoga on HR. The six included studies involved 345 and 308 participants in the intervention and control group respectively. The random effects model was chosen for the overall effect analysis because of a high variance (I^2^ = 96.0%, *p* < 0.0001). The analysis showed a that HR significantly decreased by 2.55 beats/min (z = −2.77, *p* = 0.006).

Practice of Yoga has also shown to have a significant effect on the respiration. The top themes highlighting the effect of Yoga were on tidal volume (TV), vital capacity (VC), Forced vital capacity (FVC), Forced expiratory volume (FEV1), and peak expiratory flow rate (PEFR). While these parameters do not show a significant change in control group, they all show a significant increase after yoga [23,25,26,35]. Only Akhani et al., 2019 [25] did not find a significant increase in TV.

#### Other physiological effects

Besides cardiovascular and respiratory parameters, yoga effects other physiological parameters as well. It was found that yoga decreases serum IFN-γ levels [30]. While serum cortisol levels increase after yoga [30], salivary cortisol is shown to decrease [39]. Moreover, yoga not only significantly reduces PMS symptoms, but it also works better than calcium carbonate [32].

### Psychological impact of yoga

**Figure 4.**
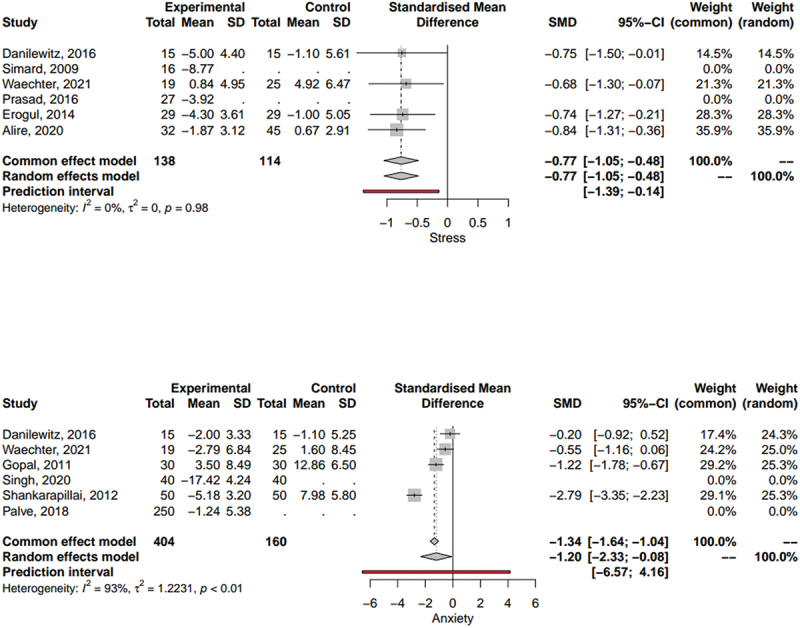
The Meta-analysis on the Effect of Yoga on Stress (4A) and Anxiety (4B) Among Medical and Dental Students.

The effect of yoga on the psychology of medical and dental students has also been observed. Figure 4A summarizes the effects of yoga intervention on stress. Data from 4 studies was with more than 100 participants in treatment and control groups was included, yielding a significant overall effect of a stress reduction of 0.77 on standardized assessments due to the yoga intervention (z = 5.29, *p* < 0.0001). Both the random effects model and common effect model yielded similar results (I^2^ = 0.0%, *p* = 0.98). Similarly, Figure 4B represents the effect of Yoga on anxiety. 4 studies with 114 and 160 participants in the intervention and control groups were included. The results showed significant (z = −2.52, *p* = 0.01) reduction of 1.2 in standardized anxiety tests in intervention group compared to the control. Due to a high variance (I^2^ = 92.9%, *p* < 0.0001), random effects model was chosen for the overall effect analysis. Besides anxiety and stress, depression was also observed to significantly decrease in yoga group [43,47,49,55,57].

### Other psychological parameters:

Yoga has also been shown to improve other cognitive aspects of medical and dental students. For instance, practice of yoga has been shown to enhance memory and attention [53,58,60,61,63]. However, Kumar et al., 2020 [43] found no significant change in attention or memory. Yoga also enhances self-regulation and state-mindfulness [33,50,62]. Other advantages of yoga include better sleep quality [11], reduced depression [45,49,53,55,56], and reduced pain [37]. Overall, yoga enhances the mental wellbeing of medical and dental students [45].

### Yoga practice and academic improvement

The impact of yoga on medical and dental students’ academic performance were investigated. Only 6 articles focused on this aspect. Most studies found an increase in academic performance following yoga practice [28,30,58,64] which could be due to a better sleep quality [28], improved attention and mental focus [58], or reduction in stress associated with examination [30]. On the other hand, Lee et al.. (2022) [40] and Waechter et al.. (2021) [43] found no evidence of academic improvement following yoga.

## Discussion

Yoga, an ancient Hindu spiritual practice in India has been evolving over thousands of years. It includes physical exercise such as asanas, pranayama and kriyas, breath control, meditation, with a primary focus on an all-inclusive role where the person achieves balance and harmony in the body and mind in totality [69] which may enhances physical, mental, intellectual, and spiritual well-being [70].

There has been a growing interest in the potential benefits of yoga on both physiological and mental well-being considering its easy access and the non-invasive way to prevent and manage the disease burden. Practicing yoga is affordable and accessible to many people because of the plausible positive impact on health and cognitive outcomes. Therefore, growing interest in the use of yoga to manage or treat disease burden has been a topic of research among academicians. Several ongoing randomized clinical trials have provided preliminary evidence of positive psychosomatic impact of yoga in preventing and managing diseases.

Healthcare trainees often face high levels of stress, anxiety, and burnout due to the rigorous demands of their studies and training. Consequently, exploring the impact of yoga on medical and dental students has become a subject of interest in recent years. This review aims to assess the existing literature and shed light on the potential positive effects of incorporating yoga into the lives of medical and dental students to improve their physical and psychological well-being as well as overall academic performance.

Our data from the 3826 medical and dental students found that yoga intervention improves physiology outcomes such as cardiovascular system (CVS) and respiratory system. In addition, there is also a significant improvement in relieving stress, anxiety and depression which may enhance the academic and social life of the medical and dental students. This study is the first meta-analysis to investigate the efficacy of yogic intervention on cardiopulmonary functions and psychological wellbeing in medical and dental students.

## Impact of yoga on physiology

A fundamental aspect of yoga is the practice of various asanas (postures), which involve stretching and strengthening different muscle groups. Recent studies have shown that regular yoga practice may increase the flexibility and balance in male college athletes and therefore, a way to improve athletic performance [71]. Increased flexibility can also reduce the risk of injuries and promote better body posture and alignment among healthy individuals. Specific yoga practices often incorporate mindful breathing techniques, such as pranayama, which involve controlled breath regulation. A retrospective case-control study showed the antihypertensive effect of yoga and overall improvement of cardiovascular function amongst general patient population [72].

Our findings revealed that yoga has a positive role in reducing inflammatory markers such as serum interferon-gamma (IFN-γ) levels [30] amongst medical and dental students. A recent review also highlights the potential for yoga to be used as adjuvant therapy in conditions with an inflammatory component, such as cardiovascular and chronic respiratory disease, by reducing levels of C-reactive protein, interleukin-1, interleukin-6, tumor necrosis factor-alpha (TNF-α) and IFN-γ [73].

Our systematic-review also showed that serum cortisol levels increase after yoga amongst medical trainee [30]. In contrast, we also found evidence that salivary cortisol levels decrease following yoga [39]. This discrepancy may be attributed to various factors, with a reduction in stress levels being a likely significant influence, leading to decreased cortisol levels. The interplay between reduced stress and other variables, such as inflammation, might cause either an increase or decrease in serum and salivary cortisol levels. These contradictory effects underscore the complexity of the relationship and highlight the need for further targeted research to elucidate the underlying mechanisms.

## Yoga and CVS

Cardiovascular diseases are the leading cause of mortality worldwide [60,74]. Based on WHO data from 2019, an estimated 17.9 million people died from cardiovascular diseases, representing 32% of all global deaths. Of these deaths, 85% were due to heart attack and stroke [75]. Furthermore, cardiovascular diseases are more prevalent in developing countries like India as compared to their western counterparts [76,77]. Hence, it is important to understand that simple therapeutic strategies such as practicing yoga are cost-effective and yield promising results in reducing the disease burden and mortality rate. Several studies have examined the positive effect of yoga on physiological, biochemical, and neuro-cognitive parameters [78–80].

Our study, through a meta-analysis, substantiates that engaging in several weeks of yoga practice can enhance the CVS among medical and dental students. This is highlighted by reductions in heart rate (HR), systolic blood pressure (SBP), and diastolic blood pressure (DBP). The mechanisms behind yoga’s improvement of CVS health are multifaceted and may include activation of the parasympathetic system, mitigation of stress levels, or enhancements in overall fitness. A randomized controlled trial further supports yoga’s role in cardiovascular health by normalizing body mass index, SBP, and both low and high-density lipoproteins [81]. The specific impact of yoga on CVS might also vary according to the type of practice. For instance, the ‘Brahmari Pranayama’ technique is particularly effective in lowering blood pressure and HR, promoting a state of rest and improved digestion by fostering parasympathetic dominance within the body [82,83]. Overall, the findings of this study offer compelling evidence for the potential advantages of yoga in maintaining cardiovascular health for medical and dental students.

### Yoga and SBP/DBP

Our findings indicate that yoga leads to a significant reduction in SBP and DBP among medical and dental students. This observation could be attributed to various factors, including a decrease in heart rate (HR), reduction in stress levels, and overall improvement in fitness. Our results are consistent with prior research, demonstrating that yoga practice can significantly enhance various health metrics, including SBP, DBP, HR, respiratory rate, waist circumference, waist-to-hip ratio, lipid profile, HbA1c, and insulin resistance [81].

This discovery may hold significant value for medical and dental students, as it points to a potential decrease in the risks of coronary heart disease and stroke. Research has suggested that lowering SBP by just 3 mmHg could reduce stroke mortality by 8% and cut the incidence of coronary heart disease by 5% [84,85]. Moreover, a recent meta-analysis has emphasized the impact of yoga, revealing a substantial reduction in both SBP and DBP by approximately 4.7 and 3.2 mm Hg, respectively, contributing significantly to the prevention of cardiovascular diseases [86].

### Yoga and HR

Our findings, which show a notable reduction in HR and blood pressure after weeks of Common Yoga Protocol practice, align with prior studies suggesting that yoga effectively lowers blood pressure [87,88]. Yoga interventions induce para-sympathomimetic effects, leading to reductions in SBP, DBP, as well as HR [89,90]. This study did not find any significant difference in systolic-diastolic BP in the comorbid group as compared to the naïve groups. Although the cause could not be ascertained, a potential reason could be the diminished vasodilatory effects resulting from reduced nitric oxide in compromised endothelium. Earlier studies by Chauhan et al. [91] among others had reported similar effects of 1-month yoga and meditation practice in the reduction of SBP, DBP and body mass index (BMI).

## Yoga and respiratory system

To assess the physiological impact of yoga on respiratory functions, various lung function tests are employed. Spirometry is a common test that measures lung volumes and capacities, including forced vital capacity (FVC) and forced expiratory volume (FEV1). These metrics indicate the amount of air a person can exhale forcibly and rapidly after a deep inhalation. Improvement in these values signifies enhanced lung function and efficiency. Peak expiratory flow rate (PEFR) is another measure used to assess the maximum speed at which air can be exhaled forcefully, reflecting airway patency.

In our present study focusing on respiratory parameters, the majority of the articles we included emphasized the impact of yoga on several aspects, including tidal volume (TV), vital capacity (VC), FVC, FEV1, and PEFR. Our analysis revealed that these parameters generally saw a significant increase following yoga practice in the majority of the studies [23,25,26,35]. However, one particular study, Akhani et al., 2019 [25], did not identify a substantial increase in TV.

Our findings are in alignment with previous research, adding to the growing body of evidence supporting the positive effects of yoga on pulmonary functions. A recent meta-analysis explored the impact of yogic interventions on respiratory functions in healthy individuals and found substantial enhancements in FVC, FEV1, the FEV1/FVC ratio, PEFR and respiratory muscle strength metrics such as Maximal inspiratory and expiratory pressures [92]. Similarly, a study targeting healthy inactive middle-aged individuals showed marked improvements in both physical and respiratory functions following an 8-week yoga intervention [93]. In another compelling investigation, participants demonstrated long-term changes in their resting breathing pattern, characterized by increased TV and reduced ventilatory response to both chemoreceptor and mechanical stimuli [94]. These findings underscore the idea that consistent engagement in yoga’s breathing exercises can influence innate respiratory regulation, further supporting the therapeutic potential of yoga in respiratory health.

Yoga’s positive impact on respiratory functions can be attributed to a combination of physiological mechanisms. Engaging in yoga practices involves conscious regulation of breath, known as pranayama, which enhances the efficiency of the respiratory system. Deep and controlled breathing techniques, such as Anulom Vilom and Kapalbhati Pranayama, help to strengthen the respiratory muscles, increase lung capacity, and improve the exchange of oxygen and carbon dioxide [95,96]. These practices stimulate the parasympathetic nervous system, leading to relaxation and decreased respiratory rate. Furthermore, chest expansion and stretching, enhances chest mobility, allowing for deeper inhalation and exhalation. The act of maintaining poses also engages the respiratory muscles, providing a mild form of resistance training for these muscles [97].

Chemoreceptors play a crucial role in regulating the respiratory center, which is responsible for controlling the rate and depth of breathing. Located primarily in the carotid bodies in the carotid arteries and the aortic bodies in the aorta, these specialized sensory cells are sensitive to changes in the levels of certain gases, particularly oxygen (O_2_) and carbon dioxide (CO_2_), as well as the pH of the blood. When oxygen levels in the blood decrease (hypoxia) or carbon dioxide levels rise (hypercapnia), the chemoreceptors detect these changes and send signals to the respiratory center in the brainstem. These signals trigger adjustments in the rate and depth of breathing to restore proper gas exchange. In response to low oxygen levels, chemoreceptors stimulate an increase in respiratory rate and depth, helping to improve oxygen intake. Similarly, in the presence of excess CO_2_, chemoreceptors prompt the respiratory center to initiate faster and deeper breathing to expel CO_2_ and maintain proper acid-base balance. The interaction between chemoreceptors and the respiratory center is a vital feedback loop that ensures the body’s respiratory rate matches its metabolic demands and maintains blood gas levels within a narrow range. This regulatory mechanism helps ensure sufficient oxygen supply and efficient removal of CO_2_, contributing to overall respiratory homeostasis [98].

Various yoga practices are based on specific breathing techniques that contribute to the process of eliminating CO_2_ from the body through its unique pattern of forceful exhalations and passive inhalations. This technique can help enhance respiratory efficiency and facilitate the removal of CO_2_ from the body. Kapalbhati involves rapid, forceful exhalations achieved by contracting the abdominal muscles. This active exhalation expels a significant amount of air from the lungs, leading to a more efficient removal of CO_2_. Exhaling vigorously leads to a larger volume of CO_2_-rich air pushed out of the lungs, creating space for fresh, oxygen-rich air to be inhaled during the subsequent passive inhalation. The forceful exhalations also require the engagement of the diaphragm, abdominal muscles, and intercostal muscles. Regular practice strengthens these respiratory muscles, improving their efficiency in moving air in and out of the lungs. Adequate oxygen levels are essential for cellular metabolisms, which in turn produce CO_2_ as a waste product that needs to be eliminated. Additionally, Kapalbhati, like other pranayama techniques, helps reduce stress and anxiety. Stress can contribute to shallow breathing, where not enough air is exchanged, leading to an accumulation of CO_2_ in the body. By promoting relaxation and deep, controlled breathing, Kapalbhati helps counteract shallow breathing patterns and ensures the efficient elimination of CO_2_ [99].

These findings are promising, revealing that yogic interventions significantly enhance pulmonary functions and respiratory muscle strength parameters in physically healthy individuals. Yoga’s influence on respiratory functions is a multi-faceted process involving controlled breathing techniques and specific asanas. The combined effect of these practices enhances lung capacity, strengthens respiratory muscles, and promotes relaxation, ultimately leading to improved respiratory efficiency. This can potentially benefit individuals with respiratory conditions such as asthma, and other respiratory diseases [100,101].

## Impact of yoga on psychological well being

Numerous independent studies and reviews have collectively demonstrated that yoga serves as an efficacious remedy for managing stress and anxiety [70,102,103]. College students often have low levels of physical activity and thus ample research has been conducted to analyze the effect of yoga on students’ mental health. The data from these studies indicate that yoga has beneficial effects at the psychophysiological level, leading to reduced stress levels in students and an overall enhancement in well-being [104–107]. Not all students will have the requisite skills to manage stress and employ effective coping strategies. Among these strategies, physical activity is one that might be underutilized by a considerable number of students.

Our meta-analysis, which included over 100 medical and dental students in both treatment and control groups, showed a significant overall effect of stress reduction as a result of the yoga intervention, as measured by standardized assessments [43,46,47,51,54,57]. Similarly, studies focusing on the effect of yoga on anxiety, with sample sizes of 114 and 160 participants in the intervention and control groups respectively, demonstrated a significant reduction in anxiety as measured by standardized tests [30,43,44,48,55,57]. In addition to the positive impacts on stress and anxiety, our systematic-review found a notable decrease in depression among those participating in the yoga group [45,49,53,55,56].

Our analysis also revealed that yoga appears to have a positive effect on overall intelligence, visuospatial working memory, and attention among medical students [58–60,63]. Additionally, research has demonstrated that the practice of yoga can enhance fluid intelligence, general mental abilities, and executive functions [108,109].

The mechanisms underlying the positive impact of yoga on mental health are complex and multifaceted. The proposed mechanisms by which yoga might have an impact on mental health include cognitive/affective and biologic mechanisms [110].

The cognitive mechanism of yoga includes directing attention to the present moment, encompassing thoughts, feelings, and body sensations without judgment. Yoga fosters mindfulness and self-awareness, allowing individuals to observe their thoughts and emotions without judgment. Our analysis revealed that medical and dental students who engage in yoga practice demonstrate improved mindfulness and self-awareness [53,56,57,62]. Therefore, this practice can lead to enhanced emotional regulation and reduced reactivity to stressful situations.

By integrating this daily practice of mindfulness, students foster the ability to concentrate on current experiences rather than dwelling about the past or future. Moreover, the non-judgmental approach helps alleviate self-criticism. By learning to attend to their immediate experiences, including thoughts and emotions, individuals realize calmer and more centered state of mind thereby decreasing stress and anxiety while promoting mental health.

Biologic mechanisms of yoga also affect the underlying pathophysiology of depression and anxiety. Yoga practices have been associated with positive effect on the structure and/or function of the brain areas such as hippocampus, amygdala, prefrontal cortex, cingulate cortex, and brain networks [111]. Yoga-based practices also regulate the autonomic nervous system (ANS), which is linked to depression and anxiety [112,113]. Yoga improves the ANS balance by promoting the activation of the parasympathetic nervous systems (PNS), responsible for the body’s rest-and-digest response, while inhibiting the sympathetic nervous systems (SNS) responsible for the fight-or-flight response. This balance may explain yoga’s stress-reducing effects.

Beside activating PNS, yoga also increases GABA levels by stimulating the vagus nerve, leading to mood enhancement. It has also been shown that yoga can modify neurotransmitters such as serotonin, norepinephrine, dopamine, and melatonin. As neurotransmitter levels offer reliable indicators for assessing psychological conditions, these findings provide a profound understanding of the beneficial impacts that complementary therapies like yoga and meditation can have on the human body. These effects may be linked to enhanced neuroplasticity and reduced stress, both of which can influence cognitive performance positively. Additionally, yoga might reduce hypothalamic-pituitary-adrenal axis activation and inflammation, potentially impacting the underlying causes of depression and anxiety [114–116]. Regular yoga practice may thus help regulate the stress response and reduce cortisol levels, contributing to improved mental well-being.

The scientific evidence supporting the positive impact of yoga on both physiology and mental health is continually growing. Regular practice has been associated with improved flexibility, cardiovascular health, and respiratory function in both the general public as well as medical and dental students. Moreover, yoga’s stress-reducing effects and its ability to enhance mindfulness and emotional regulation make it a valuable tool for promoting mental well-being and managing various mental health conditions amongst healthcare trainees. While yoga can complement traditional medical treatments, it is essential to acknowledge that it may not be a standalone solution for severe mental health disorders. As research in this field progresses, further insights into the specific mechanisms of action and the optimal implementation of yoga as a therapeutic intervention will likely emerge, solidifying its role in enhancing overall health and well-being.

## Academic life and yoga curriculum

The impact of yoga on the academic performance of students is gaining recognition as an influential factor in their overall scholastic journey. Engaging in regular yoga practice has been linked to improvements in various cognitive and emotional aspects that directly contribute to academic success amongst medical and dental students [28,30,58,64]. The positive effect of yoga on medical and dental students’ academic performance could be attributed to enhanced mental ability, reduced stress, and better sleep quality.

Yoga’s emphasis on mindfulness and focused breathing techniques cultivates heightened concentration and cognitive clarity, enabling students to absorb and process information more effectively. Yoga enhances overall intelligence, visuospatial working memory, and attention, in medical students [58–60,63]. It has also been shown that yoga enhances fluid intelligence, general mental ability, and executive function [108,109]. The observed enhancements in general intelligence, visuospatial working memory, and attention are anticipated to have a positive impact on students’ academic accomplishments.

Additionally, the stress-reduction benefits of yoga play a significant role in enhancing mental resilience, minimizing performance anxiety, and promoting a positive mindset [30,45]. One study conducted to analyze the impact of yoga on academic stress in adolescents (ages 14 to 16) during their career-deciding exams showed that yoga effectively reduced psychosocial stress in students and could be useful in school curricula [117]. Nonetheless, it’s important to note that these protective effects might not persist if yoga practice is halted.

The physical postures and relaxation techniques incorporated in yoga also contribute to overall physical health such as better sleep quality, indirectly supporting mental acuity and attentiveness in the classroom [28]. As the evidence accumulates, educational institutions are increasingly recognizing yoga’s potential to positively impact students’ academic achievements and are incorporating it into their wellness programs to provide students with holistic support for their academic endeavors.

## Knowledge and awareness of yoga

Incorporation of beneficial evidence-based complementary practices like yoga into a student’s daily life depends on the effective delivery of such healthy practices. Achieving this goal involves scrutinizing the health-seeking behavior of the population, particularly focusing on the knowledge – practice gap. This gap refers to the discrepancy between knowing about beneficial health practices and actually adopting them. The students across the many streams are generally stressed during the academic curriculum, more specifically during the exams, seminars, and training. The knowledge and awareness about yoga play a significant role and benefit the students’ academics and quality of life. Yoga’s acceptability varies among individuals and therefore, conducting a university-wide survey and collecting feedback becomes imperative to comprehensively comprehend the knowledge – practice gap [118].

The perception of yoga’s effectiveness among medical and dental students may influence their commitment to engaging in this practice. Seven studies examined students’ awareness, attitudes, and understanding of yoga, revealing that over 90% recognize its benefits [65,65,67,68]. In contrast, fewer than 20% of students regularly practice yoga [46,47,65,66,68], citing time management issues, lack of motivation, and laziness as primary barriers [66,67]. Encouragingly, one study found that 95% of participants expressed a desire to practice yoga after participating in the study [43], while another revealed that more than 90% believe yoga should be integrated into medical school curricula to support physical and mental well-being [65].

The high awareness of yoga’s benefits among medical and dental students, coupled with a strong desire to practice it, underscores its potential value in their education. Despite barriers such as time management and motivation, most students believe yoga should be part of the curriculum. Integrating yoga into medical and dental school programs could not only support students’ physical and mental well-being but also align with their expressed preferences and beliefs.

## Potential barriers and prospects

Implementing yoga programs in medical education faces several challenges and barriers that warrant careful examination. One prominent obstacle is the need for a paradigm shift in the traditional medical curriculum, which may be resistant to integrating complementary and alternative practices like yoga. Skepticism among faculty members and administrators regarding the scientific basis and efficacy of yoga in medical training may pose a significant hurdle. Awareness among faculty and designing an appropriate faculty development program by certified yoga expert can overcome the challenges and barriers in implementing yoga in medical education. Additionally, time constraints within the medical school curriculum could be a limiting factor, as educators may perceive the incorporation of yoga as an additional demand on an already tight schedule. Infrastructure and resource limitations, such as the availability of qualified yoga instructors and suitable spaces for practice, may also impede successful implementation. Furthermore, addressing diverse student preferences, cultural sensitivities, and potential misconceptions about the spiritual aspects of yoga presents a nuanced challenge. Overcoming these barriers necessitates collaborative efforts among medical educators, administrators, and the broader medical community to foster a more inclusive and holistic approach to medical training.

To address the challenges in implementing yoga programs within medical education, there is a pressing need for extensive, forward-looking, and diverse pilot initiatives that span multiple centers and employ various methodologies [119]. These projects should aim to discern the effectiveness of incorporating a yoga curriculum, encompassing both practical sessions and theoretical components, as a countermeasure to the adverse effects of the undergraduate medical school environment. This environment has been identified as a contributing factor to the development of depression, anxiety, and ineffective coping strategies among medical students. The proposed pilot projects should systematically explore how yoga interventions can be seamlessly integrated into the curriculum. By conducting large-scale studies across multiple centers, researchers can not only evaluate the impact of yoga on mental well-being but also identify practical solutions to overcome logistical barriers, engage faculty support, and tailor the program to diverse student preferences. These initiatives are essential for paving the way toward a more comprehensive and resilient medical education system.

## Strength and limitations

The strengths of our research include comprehensive search strategy from the various databases, independent screening, and meta-analysis of all relevant homogenous included studies. A summary synthesis of all included literature showing the effects of yoga intervention on physiology, mental health, and academic life is given. Meta-analysis of various psychosomatic outcomes provides significant association in the yoga intervention group as compared to control. Our summary table has provided the duration of yoga intervention that is likely to give significant results wherever possible. A continuous yoga practice is likely to produce significant improvements in cardiovascular, respiratory, mental health parameters along with improvement in academic performance of medical students.

There are several limitations that are identified in our study. Firstly, although the included articles measured many outcomes, the articles significantly vary in terms of methodological approaches, population selection, duration of the yoga intervention, weekly frequency of yoga, practice, and studied population, etc. These variations limit the conclusive effectiveness of yoga outcomes amongst the medical students. For each outcome included in the meta-analysis, there were less than 10 studies and hence potential publication bias was not assessed. Additionally, several different types of scales were used in different studies to measure the same outcomes such as anxiety, stress, and depression. This reduced the homogeneity of the data and reduced the number of articles in physiology and mental health functions. The articles that did not use the control group were not included in the meta-analysis, further reducing the number of the articles. The absence of specific metrics related to academic performance, the lack of a direct comparison between the yoga intervention group and a control group, and the insufficient exploration of academic stressors is a current limitation due to insufficient homogenous data availability. It will be interesting to explore this avenue further in future studies with more standardised study designs. Encouraging the incorporation of these elements in future revisions would enhance the overall quality and applicability of the yoga intervention studies.

Finally, several unpublished literatures, thesis, or any grey literature were not included in the current study, thus may limit any important information regarding our results too.

## Conclusion

Yoga presents a compelling avenue to enhance the physical and mental well-being of medical and dental students. By improving effectiveness, fostering collaboration, enhancing focus, and contributing to a more positive outlook on life, yoga offers multifaceted benefits. In an era where anxiety and depression are often addressed through psychological interventions and medications, the mind-body approach embodied by yoga offers a refreshing alternative. The current study underscores the importance for medical educators to recognize and leverage the therapeutic effects of regular yoga practice. Our findings illuminate yoga’s potential to improve cardiovascular and respiratory function, promote academic satisfaction, and reduce stress, anxiety, and depression among medical and dental students. The enhancement of overall well-being and quality of life that accompanies yoga practice further underscores its value. In conclusion, integrating yoga into medical and dental curricula can aid in creating a successful and balanced academic and professional life for students. Encouraging further exploration and adoption of yoga-based education, therefore, could be a strategic move towards fostering the well-rounded development of future healthcare professionals.

## Abbreviations

**Table:** 

Cardiovascular System	CVS
Systolic Blood Pressure	SBP
Diastolic Blood Pressure	DBP
Heart Rate	HR
Tidal volume	TV
Vital capacity	VC
Forced vital capacity	FVC
Forced expiratory volume (1 second)	FEV1
Peak expiratory flow rate	PEFR
